# The effect of proportional pricing on alcohol purchasing in two online experiments

**DOI:** 10.1111/add.16723

**Published:** 2024-12-03

**Authors:** Inge Kersbergen, Amber Copeland, Robert Pryce, Petra Meier, Matt Field

**Affiliations:** ^1^ School of Medicine and Population Health University of Sheffield UK; ^2^ Department of Psychology University of Sheffield UK; ^3^ MRC/CSO Social and Public Health Sciences Unit University of Glasgow UK

**Keywords:** alcohol, alcohol purchasing, behavioural economics, consumer behaviour, portion size, proportional pricing

## Abstract

**Background and Aims:**

Buying smaller‐sized alcohol products can reduce alcohol consumption, but larger products have better value for money, which presents a barrier to switching. We tested whether proportional pricing prompts drinkers to buy smaller alcohol products and reduce alcohol purchasing.

**Design, Setting and Participants:**

This study was an online experiment set in the United Kingdom, using hypothetical shopping tasks in which participants purchased different‐sized products presented under proportional pricing (i.e. constant price per litre throughout all sizes of the same product) or standard pricing conditions. Study 1 (comprising *n* = 210 participants) was a mixed experiment with pricing condition (proportional pricing, standard pricing; within‐subjects) and drink type (lager, red wine, vodka; between‐subjects) as manipulated factors. Study 2 (comprising *n* = 90 participants) was a within‐subjects experiment with pricing condition (proportional pricing, standard pricing) and multi‐pack type (size difference‐only, quantity‐difference only, size and quantity difference) as manipulated factors. Participants were UK adult alcohol consumers.

**Measurements:**

We measured outcome variables, including alcohol purchasing (UK units) and proportion of alcohol purchased from smaller products.

**Findings:**

Proportional pricing consistently increased the proportion of alcohol purchased from smaller products [study 1: B = 10.82, 95% confidence interval (CI) = 8.72–12.92; study 2: B = 11.64, 95% CI = 3.50–19.77], indicating a switch to smaller products. However, this did not consistently reduce the total amount of alcohol purchased among drink and product types: proportional pricing reduced the total units purchased from lager multi‐packs containing more rather than fewer products (B = −2.56, 95% CI = −4.82 to −0.30), but not from other types of lager multi‐packs or single lager products. Proportional pricing also reduced vodka purchasing (B = −3.30, 95% CI = −5.21 to −1.40), but the effect of proportional pricing on wine purchasing was moderated by hazardous drinking (B = −0.11, 95% CI = −0.17 to –0.05).

**Conclusions:**

Alcohol sales policies that require proportional pricing may reduce alcohol purchasing.

## INTRODUCTION

The serving, bottle and glass size of alcoholic drinks influence alcohol consumption [1]. People consume less alcohol if alcohol is served in smaller servings [2] or consumed from smaller bottles [3] and restaurants sell more wine when standard glassware is replaced with larger glasses [4, 5]. Given the costs of harmful alcohol consumption to individuals and society [6], it could have substantial benefits for public health if alcohol consumers switched from larger to smaller products.

Larger products tend to have a lower price per litre than smaller products. Drinkers report that this is an important reason for purchasing larger products and the relative price increase deters them from switching to smaller products [3]. Policies that require proportional pricing (i.e. applying the same price per litre for all sizes of the same product) may be an effective counter measure, as they would diminish the extent to which larger products entail value for money.

No research has been conducted on the effect of size‐related proportional pricing on alcohol purchasing or consumption. Studies of the effect of proportional pricing on the consumption of food and non‐alcoholic drinks have yielded promising but inconsistent findings [7, 8, 9, 10]. Additionally, a few studies investigated alcohol policies that are conceptually related to size‐related proportional pricing. One study showed that applying proportional pricing to alcohol multi‐packs would reduce alcohol purchasing [11]. An evaluation of the ban on multi‐buy promotions in Scotland, which required individual products within multi‐pack purchases to be sold at the same price per litre (or more) as a single product, revealed reductions in alcohol sales [12].

The current study aimed to investigate how proportional pricing influences alcohol purchasing. Given that weight status appears to moderate the effect of proportional pricing on food purchasing [9], we also investigated whether proportional pricing affected hazardous and light drinkers differently. We conducted two online experiments to test the effect of proportional pricing. First, we tested the effect of proportional pricing on the purchasing of single lager, wine and vodka products (study 1). Then, we tested the effect of proportional pricing on the purchasing of lager multi‐packs (study 2).

## METHODS

Our methods and data analysis plan were pre‐registered (study 1: https://osf.io/3kqgb; study 2: https://osf.io/vy4k9).

### Design

We used two mixed single‐session experiments. Study 1 used pricing condition (proportional pricing, standard pricing; within‐subjects factor) and drink type (lager, red wine, vodka; between‐subjects factor) as manipulated factors. Study 2 used pricing condition (proportional pricing, standard pricing) and trial type (size difference‐only, quantity‐difference only, size and quantity difference) as manipulated factors. Both studies received ethical approval from the Research Ethics Committee at the University of Sheffield.

### Participants (Table 1)

**TABLE 1:** Demographic characteristics.

	Study 1				Study 2
	Lager (*n* = 70)	Wine (*n* = 68)	Vodka (*n* = 69)	Overall (*N* = 207)	Lager multi‐packs (*n* = 90)
Gender					
Male; *n* (%)	47 (67.1%)	21 (30.9%)	35 (50.7%)	103 (49.8%)	58 (64.4%)
Female; *n* (%)	23 (32.9%)	47 (69.1%)	33 (47.8%)	103 (49.8%)	31 (34.4%)
Other; *n* (%)	0 (0%)	0 (0%)	1 (1.4%)	1 (0.5%)	1 (1.1%)
Age; mean (SD)	41.5 (13.3)	40.4 (13.5)	35.0 (12.3)	39.0 (13.3)	32.0 (11.1)
Annual household income; median category	£30 000–39 999	£30 000–39 999	£30 000–39 999	£30 000–39 999	£30 000–39 999
AUDIT; mean (SD)	9.9 (5.8)	10.6 (6.3)	12.1 (7.0)	10.9 (6.4)	10.2 (5.65)
Low risk (AUDIT < 8); *n* (%)	27 (38.6%)	25 (36.8)	21 (30.4)	73 (35.3)	32 (35.6%)
Increasing risk (AUDIT 8+); *n* (%)	43 (61.4%)	43 (63.2)	48 (69.6)	134 (64.7)	58 (64.4%)
(Drink type) consumption frequency					
Monthly or less; *n* (%)	13 (18.6%)	13 (19.1%)	16 (23.2%)	42 (20.3%)	12 (13.3%)
2–4 times a month; *n* (%)	23 (32.9%)	25 (36.8%)	36 (52.2%)	84 (40.6%)	33 (36.7%)
2–3 times a week; *n* (%)	27 (38.6%)	21 (30.9%)	13 (18.8%)	61 (29.5%)	32 (35.6%)
4 or more times a week; *n* (%)	7 (10%)	9 (13.2%)	4 (5.8%)	20 (9.7%)	13 (14.4%)
Motivation to reduce drinking; mean (SD)	3.3 (3.3)	4.2 (3.5)	3.5 (3.2)	3.6 (3.3)	3.4 (3.09)
Duration (min); mean (SD)	23.1 (9.4)	26.0 (11.6)	21.7 (8.1)	23.6 (9.9)	20.1 (7.57)

Abbreviations: AUDIT = Alcohol Use Disorders Identification Test; SD = standard deviation.

We used quota sampling to recruit participants (study 1: *n* = 210; study 2: *n* = 90) through the participant recruitment platform Prolific (https://www.prolific.com/), which has higher data quality than other recruitment platforms [13]. Participants were eligible if they consumed at least 1 UK unit per week and were aged at least 18 years. We recruited to fill quotas for self‐reported weekly alcohol consumption (equally split across: 1–13 units per week; 14+ units per week) and, for study 1, also drink type (equally split across: regular consumption of lager; red wine; vodka; see Supporting information). The sample size was based on a power calculation in GLIMMPSE [14], which showed that we needed 70 participants per drink type in study 1 and 90 participants in study 2 to have 90% power to detect a 1.5 unit (study 1) or 1.1 unit (study 2) difference between pricing conditions and an interaction between pricing condition and risky drinking where the effect of pricing condition leads to a 1‐unit greater difference for risky drinkers than low risk drinkers. The target effect size for study 2 was lower, as it was informed by study 1 results. We included attention and comprehension checks to discourage careless or inattentive responding (study 1: 3 checks; study 2: 2 checks). No participants were excluded from the analysis for failing too many attention checks, but we excluded three participants from study 1 because they reported not consuming the drink type to which they were allocated. Our final analysis sample was *n* = 207 for study 1 and *n* = 90 for study 2.

### Materials

#### Hypothetical shopping task

Both studies used the same hypothetical shopping task with minimal differences. In study 1, participants were allocated to one of three versions of the task based on whether they were regular lager, red wine or vodka drinkers. In study 2, all participants completed the same task with lager multi‐packs. Participants were asked to imagine buying the alcohol they regularly consume from a shop to consume at home later that evening. They were also instructed to imagine they had no alcoholic drinks at home, had no opportunity to go to a different shop and that the products presented to them on each trial were the only available products. These instructions were based on standardized instructions that have been successfully administered in hypothetical alcohol purchase tasks [15].

**FIGURE 1:**
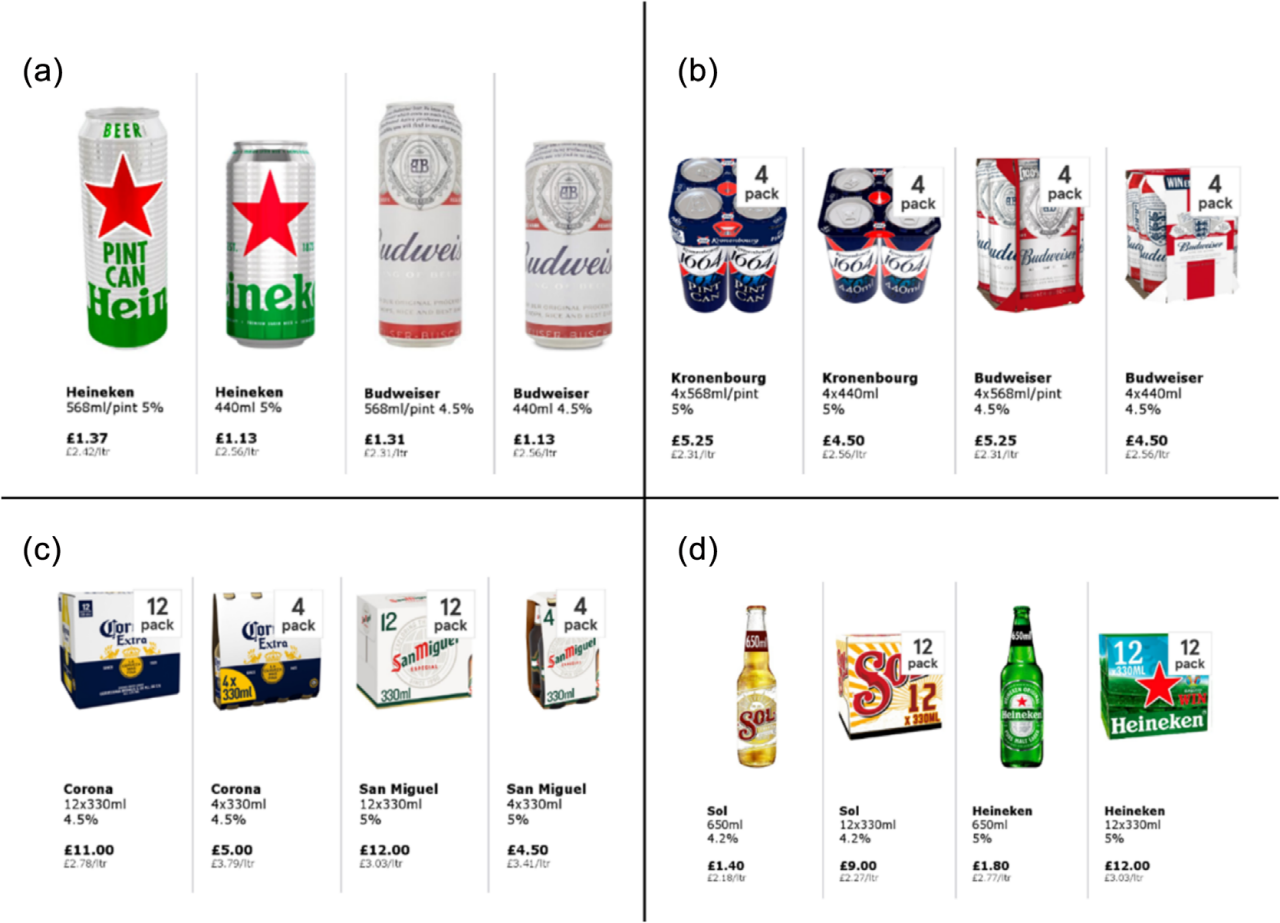
Example of images presented in lager trials in study 1 (a) and size‐difference only (b), quantity‐difference only (c), and size and quantity difference trials in study 2 (d)

In each trial, participants were shown four products. These were from two distinct brands with a smaller and larger size option for each brand. Each product was accompanied by a price label showing the product price and the price per litre (Figure 1). Study 2 used three types of trials to investigate how proportional pricing affects alcohol purchasing of products sold in multi‐packs. The first type presented products that differed only in container size, but not multi‐pack quantity (e.g. four‐pack of 568 ml cans and a four‐pack of 440 ml cans), which is equivalent to the trial type used for study 1. The second type presented products that differed only in the multi‐pack quantity, but not in product size (e.g. four‐pack of 440 ml cans and a 10‐pack of 440 ml cans). The third type presented products that differed in multi‐pack quantity and container size (a four‐pack of 568 ml cans and a 10‐pack of 440 ml cans).

In both studies, each trial was presented twice, once in the standard pricing condition (using current supermarket price per litre) and once in the proportional pricing condition using the price per litre of the smallest product of each brand (study 1) or the product with the highest price per litre of each brand (study 2)]. Most prices were sourced from a major UK supermarket (Tesco), with prices for products not available at Tesco sourced from other major UK supermarkets (Sainsbury’s and Asda). We only selected prices that were not subject to price promotions at the time. In each trial, participants indicated how many of the products they would purchase to consume. The order of trials was randomized and participants completed 60 trials in total. Participants could take a quick break every 15 trials if needed. This task took approximately 25 minutes to complete (Table 1). See Table 2 for trial characteristics.

**TABLE 2:** Trial characteristics.

	Study 1			Study 2			
	Lager	Wine	Vodka	Size difference only	Quantity difference only	Size and quantity difference	All trials
Price increase^a^ (£); mean (SD) [range]	0.23 (0.22) [0.03–0.76]	1.95 (0.81) [0.51–3.92]	4.25 (4.64) [0.27–23.48]	0.48 (0.35) [0.10–1.15]	2.68 (0.77) [1.40–4.30]	0.63 (0.63) [0.10–2.20]	1.26 (1.18) [0.10–4.30]
Size difference^b^ (ml); mean (SD)	228.7 (102.3)	469.0 (94.8)	446.7 (152.3)	128.0 (0.0)	0.0 (0.0)	224.0 (99.1)	117.3 (108.3)
Quantity difference^c^ (*n* products); mean (SD)	–	–	–	0.0 (0.0)	9.8 (2.5)	7.4 (3.2)	5.7 (4.8)

Abbreviations: SD = standard deviation.

^a^ Price increase for large products in the proportional pricing trials compared to standard pricing trials.

^b^ Size difference between the large product and small product presented within trials.

^c^ Quantity difference between the high quantity and low quantity product presented within trials.

#### Alcohol purchase task

Study 1 also included an alcohol purchase task. We adapted the alcohol purchase task [16] to explore alcohol demand indices for small, medium and large lager, wine and vodka products (depending on the drink type they were allocated for the hypothetical shopping task). Participants were asked to indicate how many products they would buy at eight incremental price points. The task took approximately 5 min to complete. This task was used to answer a related, but distinct research question and we will not report the results in this paper. See pre‐registration for more details (https://osf.io/3kqgb).

### Procedure

First, participants completed a demographic questionnaire measuring age, gender and annual household income. Then, study 1 participants completed the hypothetical shopping task and the adapted alcohol purchase task. The order of these tasks was randomized. Study 2 participants only completed the hypothetical shopping task. Finally, participants reported how often they consume lager, red wine or vodka (depending on drink type allocation), and completed a measure of hazardous drinking (Alcohol Use Disorders Identification Test; AUDIT [17]) and readiness to change drinking (readiness to change ruler [18]).

### Data analysis

#### Data preparation

We planned to use variable transformations if assumptions were violated. However, because outliers caused these violations, we excluded outliers instead. We excluded trials in which participants bought zero units and trials that were outliers (*z* < −3.29 or *z* > 3.29) for individual participants.

We calculated the number of UK units bought and the proportion of UK units bought from the smaller size or lower quantity * option in each trial as the outcome measures.

The proportion of UK units bought from the smaller size option followed a bimodal distribution. However, we still conducted the analysis as pre‐registered, because fixed‐effect estimates in linear mixed regression models are robust to this violation [19].

#### Pre‐registered analyses

We used linear mixed regression models with random intercepts and trials nested within participants to analyse the effect of pricing condition, hazardous drinking, trial type (study 2 only) and their interaction on (1) number of alcohol units purchased and (2) proportion of alcohol units purchased from the smaller size options. We conducted the analyses among all drink types in study 1 combined and for each drink type separately. We followed‐up significant interactions between pricing condition and trial type by repeating the analysis for each trial type separately.

As a sensitivity analysis, we repeated the analysis including trials in which participants did not buy any drinks. We also used linear mixed regression models with random intercepts and trials nested with participants to analyse the effect of trial order on number of alcohol units purchased. If the effect of trial order on alcohol purchasing was significant, we added trial order as a factor to the linear mixed regression model of number of alcohol units purchased to assess whether trial order moderated the effect of pricing condition.

#### Exploratory analyses

We calculated how much the price of the large product of each brand increased in the proportional pricing condition compared to the standard pricing condition. We used a linear mixed regression model with random intercepts at participant level to assess how the price difference was associated with the difference in units purchased in the proportion pricing condition compared to the standard pricing condition. We conducted this analysis on the brand level, with brands nested within trials nested within participants. In line with our main analyses, we conducted the analyses across all drink types and for each drink type separately. The random intercepts parameter was excluded from the ‘size‐difference only’ and ‘size and quantity difference’ models because it was redundant.

We also calculated price elasticities on the brand level for each trial. We summed the UK unit purchased among all participants for each brand with each trial and calculated the increase in UK units purchased under proportional pricing as a percentage of UK units purchased under standard pricing. To calculate the price elasticities, we divided this by the price increase for the large product of each brand as a percentage of the standard price. We present the median price elasticity and interquartile range (IQR).

## RESULTS

### Effect of proportional pricing on UK units purchased (Table 3; Figure 2)

**TABLE 3:** Mixed linear regression models showing the effect of proportional pricing, trial type (study 2), AUDIT scores and their interaction on alcohol units purchased. Trials were nested within individuals.

	Study 1				Study 2			
	Overall	Lager	Wine	Vodka	All trials	Size difference only	Quantity difference only	Size and quantity difference
	B (95% CI)	B (95% CI)	B (95% CI)	B (95% CI)	B (95% CI)	B (95% CI)	B (95% CI)	B (95% CI)
Intercept	11.20***(7.58, 14.83)	4.56*(0.92, 8.21)	9.08***(6.17, 11.99)	26.68***(20.31, 33.04)	14.42***(7.58, 21.27)	11.51**(4.41, 18.62)	18.56***(11.26, 25.86)	14.82***(7.80, 21.83)
Proportional pricing (reference: standard pricing)	−0.92*(−1.65, −0.20)	0.15 (−0.51, 0.81)	−0.18 (−0.89, 0.54)	−3.30***(−5.21, −1.40)	1.16 (−0.92, 3.24)	0.20 (−0.98, 1.39)	−2.56*(−4.82, −0.30)	1.14 (−1.01, 3.29)
AUDIT	0.78***(0.49, 1.06)	0.67***(0.36, 0.99)	0.42***(0.19, 0.66)	0.61**(0.16, 1.07)	0.53 (−0.06, 1.11)	0.67*(0.07, 1.27)	0.39 (−0.23, 1.02)	0.50 (−0.10, 1.11)
AUDIT × proportional pricing (reference: standard pricing)	−0.03 (−0.09, 0.02)	−0.02 (−0.07, 0.04)	−0.11***(−0.17, −0.05)	0.06 (−0.07, 0.20)	−0.12 (−0.29, 0.06)	−0.05 (−0.15, 0.05)	0.14 (−0.05, 0.33)	−0.11 (−0.29, 0.07)
Trial type (reference: size and quantity difference)								
Size difference only					−3.14**(−5.25, −1.04)			
Quantity difference only					4.01***(1.92, 6.10)			
Trial type × proportional pricing condition (reference: standard pricing; size and quantity difference)								
Size difference only × proportional pricing					−1.08 (−4.03, 1.88)			
Quantity difference only × proportional pricing					−3.83*(−6.77, −0.88)			
Trial type × AUDIT (reference: size and quantity difference)								
Size difference only × AUDIT					0.17 (−0.01, 0.35)			
Quantity difference only × AUDIT					−0.14 (−0.31, 0.04)			
Trial type × proportional pricing condition × AUDIT (reference: standard pricing; size and quantity difference)								
Size difference only × Proportional pricing × AUDIT					0.07 (−0.18, 0.31)			
Quantity difference only × Proportional pricing × AUDIT					0.27*(0.02, 0.52)			
Random effects								
Within‐cluster variance (σ^2^)	85.65	25.01	25.25	196.41	102.70	32.67	120.58	109.18
Intercept variance (τ_00_)	176.34	59.75	35.71	173.52	242.70	255.04	275.55	254.20
Intraclass correlation Coefficient	0.67	0.70	0.59	0.47	0.70	0.89	0.70	0.70
*n* Participants	206	70	67	69	90	87	90	90
*n* Observations	10 191	3592	3020	3579	4765	1574	1589	1602
Marginal *R* ^2^/conditional *R* ^2^	0.086/0.701	0.154/0.750	0.090/0.623	0.055/0.499	0.033/0.713	0.045/0.892	0.018/0.701	0.018/0.705

Abbreviations: AUDIT = Alcohol Use Disorders Identification Test; CI = confidence interval.

* *P* < 0.05,

** *P* < 0.01, and

*** *P* < 0.001.

**FIGURE 2:**
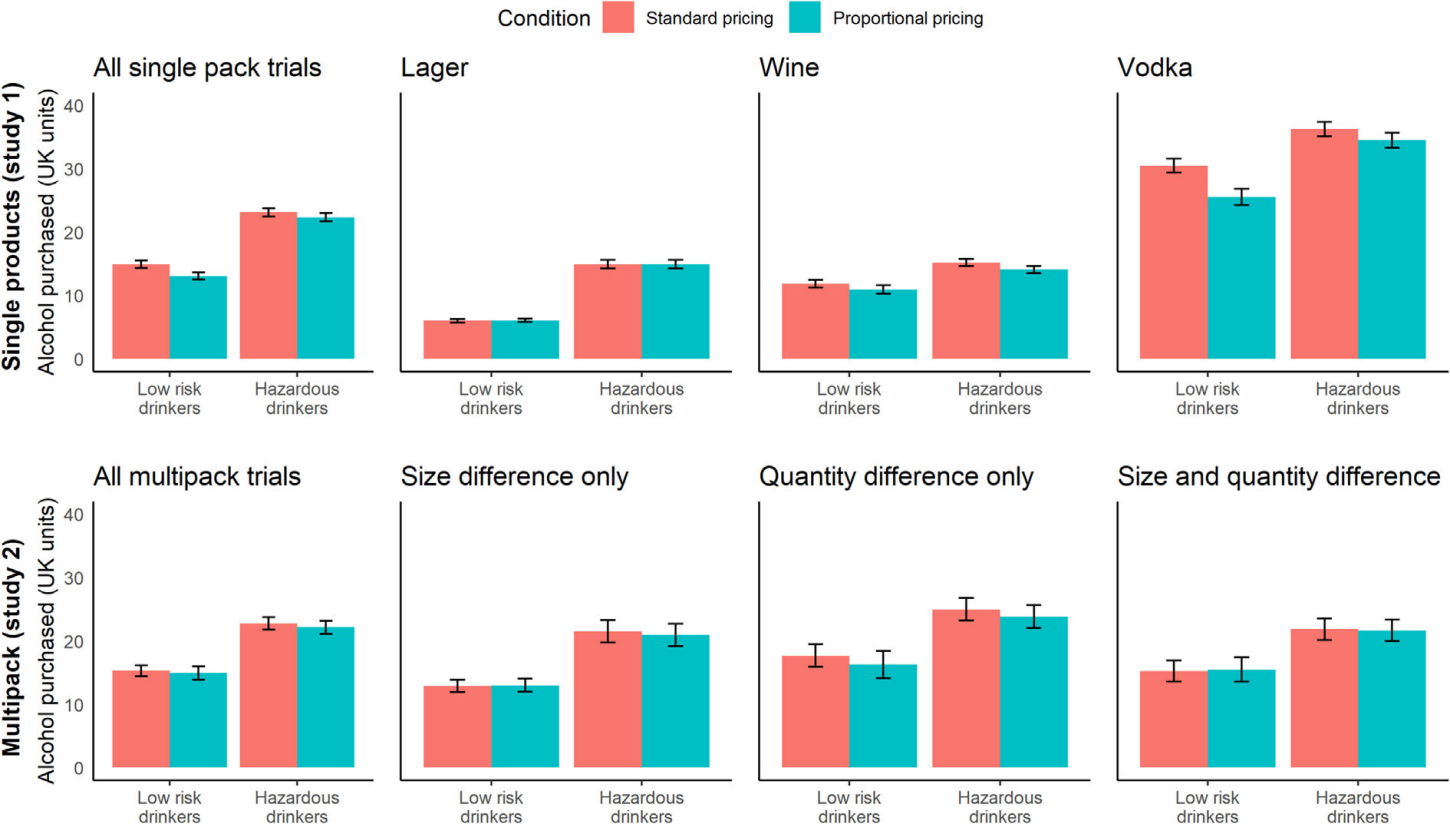
Effect of proportional pricing condition on alcohol purchasing in UK units split by low‐risk drinkers (AUDIT < 8) and hazardous drinkers (AUDIT 8+). Error bars indicate 95% confidence intervals

Among all drink types in study 1 combined, there was a significant main effect of pricing condition. Participants bought 0.9 [standard error (SE) = 0.37] fewer units in the proportional pricing condition compared to standard pricing. There was a significant interaction between pricing condition and trial type in study 2 (*P* = 0.03). AUDIT scores were positively associated with alcohol units purchased in study 1, but not study 2. The interaction between pricing condition and AUDIT scores was not significant.

The main effect of proportional pricing varied across drink types and trial types. We found no main effect of proportional pricing or interaction for purchasing of single lager products. For wine purchasing, we found no main effect of proportional pricing, but a significant interaction with AUDIT scores, which showed greater reductions in alcohol purchasing in the proportional pricing condition compared to standard pricing as AUDIT scores increased. For vodka purchasing, we found a main effect of proportional pricing, but no interaction with AUDIT scores. The separate analyses for lager multi‐pack trial types showed that proportional pricing significantly affected alcohol purchasing from trials with differing multi‐pack quantities only.

Sensitivity analyses showed that after including trials in which participants did not buy any drinks, three regression coefficients that were not significant in the main analyses reached significance (Supporting information, Table S1). There was a significant main effect of AUDIT scores on alcohol purchasing among all multi‐pack trial types (B = 0.7, SE = 0.28), a significant interaction between the quantity difference only trial type and AUDIT scores (B = −0.2, SE = 0.10) on alcohol purchasing across all multi‐pack trial types and a significant main effect of AUDIT scores on alcohol purchasing on size and quantity difference trials (B = 0.7, SE = 0.27).

Trial order did not significantly affect UK units purchased from single products, but significantly influenced UK units purchased from lager multi‐packs, with participants purchasing fewer units on later trials (B = −0.04, SE = 0.01; Supporting information, Table S2). This meant that, on average, participants purchased 2.4 fewer UK units on the last multi‐pack trial compared to the first multi‐pack trial. Trial order did not significantly moderate the effect of pricing condition on UK units purchased (Supporting information, Table S3), suggesting that randomization was effective at distributing trials throughout the trial.

### Effect of proportional pricing on proportion of units purchased from smaller products (Table 4; Figure 3)

**TABLE 4:** Mixed linear regression models showing the effect of proportional pricing, trial type (study 2), AUDIT scores and their interaction on proportion of alcohol units purchased from small products. Trials were nested within individuals.

	Study 1				Study 2			
	Overall	Lager	Wine	Vodka	All trials	Size difference only	Quantity difference only	Size and quantity difference
	B (95% CI)	B (95% CI)	B (95% CI)	B (95% CI)	B (95% CI)	B (95% CI)	B (95% CI)	B (95% CI)
Intercept	30.64***(21.51, 39.76)	43.61***(25.64, 61.57)	15.09**(3.95, 26.24)	34.46***(18.89, 50.02)	50.59***(38.62, 62.57)	49.60***(32.94, 66.26)	58.30***(41.73, 74.87)	50.54***(40.88, 60.20)
Proportional pricing (reference: standard pricing)	10.82***(8.72, 12.92)	4.54**(1.57, 7.51)	10.67***(7.19, 14.15)	19.91***(15.54, 24.27)	11.64**(3.50, 19.77)	3.49 (−1.70, 8.68)	13.25***(7.06, 19.44)	11.70*(2.54, 20.87)
AUDIT	−0.50 (−1.22, 0.22)	−1.21 (−2.78, 0.35)	−0.22 (−1.12, 0.68)	−0.29 (−1.40, 0.83)	−0.57 (−1.59, 0.45)	−1.65*(−3.06, −0.24)	−1.17 (−2.58, 0.25)	−0.61 (−1.43, 0.21)
AUDIT × proportional pricing (Reference: standard pricing)	−0.22**(−0.39, −0.06)	0.05 (−0.20, 0.30)	−0.41**(−0.69, −0.13)	−0.53***(−0.84, −0.23)	−0.17 (−0.86, 0.51)	0.24 (−0.20, 0.67)	−0.39 (−0.92, 0.13)	−0.18 (−0.95, 0.59)
Trial type (reference: size and quantity difference)								
Size difference only					0.13 (−8.09, 8.35)			
Quantity difference only					7.54 (−0.64, 15.72)			
Trial type × proportional pricing condition (reference: standard pricing; size and quantity difference)								
Size difference only × Proportional pricing					−8.04 (−19.59, 3.51)			
Quantity difference only × Proportional pricing					2.20 (−9.31, 13.71)			
Trial type × AUDIT (reference: size and quantity difference)								
Size difference only × AUDIT					−1.14**(−1.83, −0.46)			
Quantity difference only × AUDIT					−0.59 (−1.28, 0.10)			
Trial type × proportional pricing condition × AUDIT (reference: standard pricing; size and quantity difference)								
Size difference only × proportional pricing × AUDIT					0.40 (−0.57, 1.37)			
Quantity difference only × Proportional pricing × AUDIT					−0.25 (−1.22, 0.73)			
Random effects								
Within‐cluster variance (σ^2^)	724.00	505.09	599.62	1035.33	1570.05	626.92	904.91	1988.03
Intercept variance (τ_00_)	1108.34	1457.80	513.21	1043.86	596.01	1348.29	1385.01	270.86
Intraclass correlation Coefficient	0.60	0.74	0.46	0.50	0.28	0.68	0.60	0.12
*n* Participants	206	70	67	69	90	87	90	90
*n* Observations	10 191	3592	3020	3579	4765	1574	1589	1602
Marginal *R* ^2^/conditional *R* ^2^	0.018/0.612	0.027/0.750	0.016/0.470	0.029/0.516	0.052/0.313	0.041/0.696	0.034/0.618	0.018/0.135

Abbreviations: AUDIT = Alcohol Use Disorders Identification Test; CI = confidence interval.

* *P* < 0.05,

** *P* < 0.01, and

*** *P* < 0.001.

**FIGURE 3:**
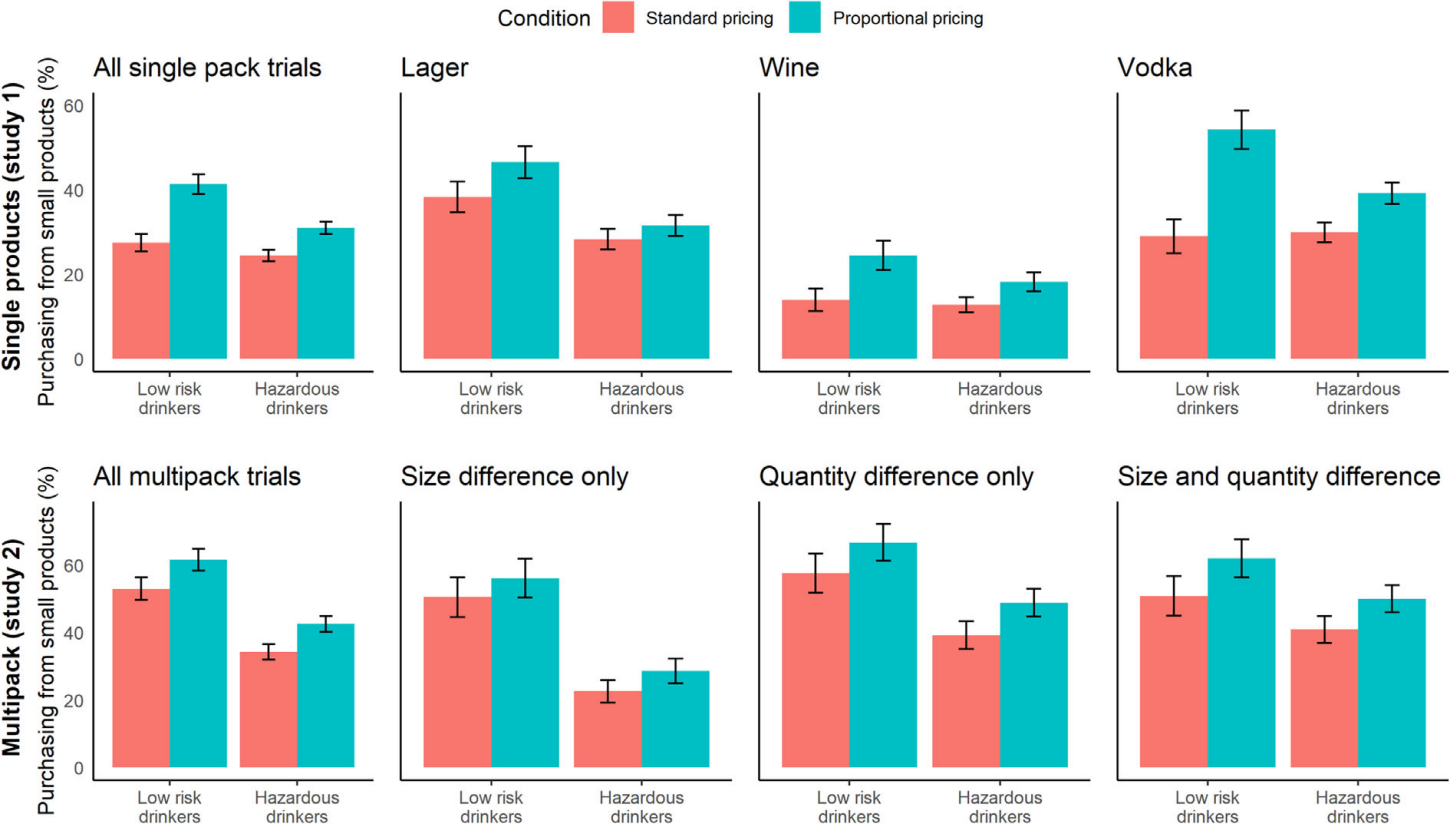
Effect of proportional pricing condition on the proportion of UK units purchased from small products split by low‐risk drinkers (AUDIT < 8) and hazardous drinkers (AUDIT 8+). Error bars indicate 95% confidence intervals

Among all drink types, participants bought a greater proportion of their UK units from smaller products in the proportional pricing condition compared to the standard pricing condition (study 1: an increase of 10.8 percentage points; SE = 1.07; *P* < 0.001; study 2: an increase of 11.6 percentage points; *P* = 0.005). AUDIT scores were not significantly associated with the proportion of UK units bought from smaller products. The interaction between AUDIT scores and pricing condition was significant, and showed that the effect of pricing condition was reduced as AUDIT scores increased.

The separate analyses for each drink type showed the same pattern of results, except for single and multi‐pack lager products, which did not show an interaction between pricing condition and AUDIT scores, and multi‐pack trials that only varied the product size, which did not show a main effect of pricing condition and did show significant associations with AUDIT scores.

### Association between change in price and purchasing (Supporting information, Table S4; Figure 4)

**FIGURE 4:**
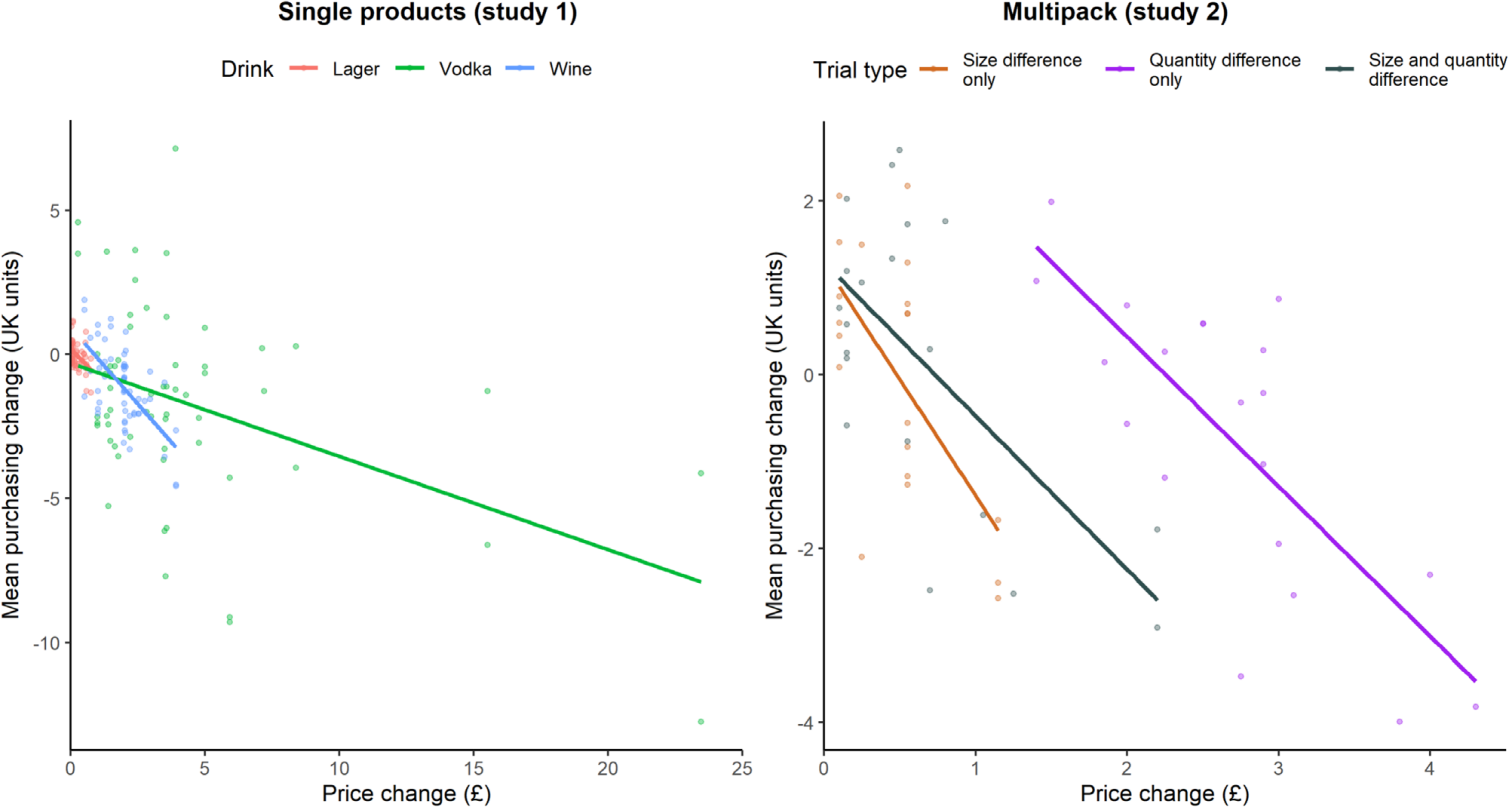
Scatterplot of the association between the price difference between the standard pricing condition and proportional pricing condition and the mean purchasing change between the standard pricing and proportional pricing condition for each trial. Linear fitted lines are plotted for the different drink types (study 1) and trial types (study 2) separately

The association between price increase and reduction in units purchased in the proportional pricing condition was significant among all drink types, with a 0.34 UK unit reduction (SE = 0.03) in alcohol purchasing for each £1 increase in the price difference between proportional and standard pricing. The association was also significant for each drink type separately, with a 0.95 UK unit reduction for lager (SE = 0.25; median price elasticity = −0.03; IQR = −0.41 to 0.67), a 1.04 UK unit reduction for wine (SE = 0.12; median price elasticity = −0.64; IQR = −1.15 to −0.30) and a 0.31 UK unit reduction for vodka (SE = 0.05; median price elasticity = −0.70; IQR = −1.17 to −0.06) for each £1 increase. The association was also significant for each multi‐pack trial type, with a 2.7 UK unit reduction for size difference only trials (SE = 0.60; median price elasticity = 0.71; IQR = −0.92 to 3.10), a 1.7 UK unit reduction for quantity difference only trials (SE = 0.34; median price elasticity = −0.11; IQR = −0.66 to 0.30) and a 1.8 UK unit reduction on size difference and quantity difference trials (SE = 0.39; median price elasticity = 0.38; IQR = −0.50 to 3.34) for each £1 increase.

## DISCUSSION

The present study aimed to test the effect of proportional pricing on alcohol purchasing. Proportional pricing increased the proportion of alcohol purchased from smaller products. This effect was stronger in lighter, compared to heavier, drinkers. This suggests that once the value for money consideration is removed, consumers may prefer smaller products. Switching to smaller products is not an inevitable outcome from the task design, as participants who purchased large products under standard pricing could also have purchased the exact same products (as proportional pricing does not give smaller products a price advantage either), switched to the other brand within the trial and purchased the same amounts of large products or purchased fewer large products.

Despite the partial switch to smaller drinks, proportional pricing did not consistently influence alcohol purchasing among all drink types. Proportional pricing led to reductions in the total units purchased from lager multi‐packs with more compared to fewer products, but not from other types of lager multi‐packs or single lager products. Proportional pricing also led to reduced vodka purchasing, but the effect of proportional pricing on wine purchasing was moderated by hazardous drinking. This suggests that price and product size may have unique effects on purchasing of different types of drinks.

As proportional pricing increased the proportion of alcohol purchased from smaller products, one possible explanation for this finding could be the absolute price difference between the standard pricing and proportional pricing conditions for the different drink types. As shown in Table 2, large vodka products on average cost £4.61 more under proportional pricing, whereas large wine products cost £2.00 more and large lager products cost only £0.23 more. Additionally, lager multi‐packs with a greater number of products on average cost £2.75 more under proportional pricing, whereas the cost of the other lager product types increased by less (up to an average of £0.63 for multi‐packs that differed in size and quantity). Therefore, it is possible that the price difference for vodka products and large lager multi‐packs is so large that proportional pricing causes all drinkers to purchase less, whereas the smaller price difference for wine may be large enough to affect heavier drinkers who consume wine more often, but not lighter drinkers who consume wine less often. The price difference for most lager products might not be sufficient to lead to reduced alcohol purchasing. This is in line with previous research that found no effect of proportional pricing on cheap soft drinks [8]. Our explanatory analysis showing that the reduction in UK units purchased under proportional pricing increased as the price difference between standard and proportional pricing increased suggests that proportional pricing might be particularly effective for brands with a large price difference between larger and smaller products. However, as these were unplanned, exploratory analyses, this conclusion should be taken with caution and future confirmatory experiments are needed to support this hypothesis.

A main methodological strength of this study is that we presented participants with a variety of product choices. Unlike previous proportional pricing research, which only presented a few options [7, 8, 9], this meant that participants’ dislike of particular brands within the experiment did not substantially affect our overall analysis. Additionally, we presented multiple brand choices within a trial, allowing participants to switch to a potentially less preferred brand rather than purchasing their preferred brand at a higher price point or switching to smaller products.

Our study also had limitations. First, hypothetical purchasing may not reflect real‐life purchasing or consumption. For example, participants may not have intended to consume all products they purchased in one sitting, but to save leftovers for later (e.g. intending to consume most, but not all, of a selected bottle of wine). However, previous research showed that hypothetical purchasing is highly correlated with alcohol consumption under controlled laboratory conditions [20] and our findings are therefore likely to reflect actual consumption. Additionally, having smaller bottles of wine in the home was associated with reduced consumption [3]. Therefore, even for products that people may not intend to finish in one setting (e.g. wine and spirits), purchasing smaller products probably reduces overall consumption. Nevertheless, future research should investigate the direct effect of proportional pricing on actual alcohol consumption in participants’ usual drinking settings. Secondly, we do not know how other factors that influence purchasing decisions (e.g. the possible inconvenience of carrying multiple small products instead of a single large product, or the drinking occasion they are purchasing for) moderate the proportional pricing effect. While the within‐subject experimental design means that individual differences in the imagined occasion are equally applied to the standard and proportional pricing conditions, they might explain some observed differences between drink types, if the imagined occasion type differed fundamentally between lager, wine and vodka. Further research, particularly using real‐life experiments, is needed to understand how such additional decision parameters moderate the effect of proportional pricing. Thirdly, we did not allow participants to switch between different drink types. Therefore, it is unknown whether proportional pricing might lead to consumers switching to a cheaper drink type (e.g. switching from wine to lager) rather than to smaller products of the same type. Finally, our study investigated the effect of increasing the cost of the drink with the lowest price per litre. However, retailers might also reduce the cost of the drink with the highest price per litre in response to a proportional pricing policy. Our study is unable to assess whether this response might result in increased alcohol purchasing.

To conclude, this experiment showed that proportional pricing may reduce vodka and wine purchasing and purchasing from large lager multi‐packs. Proportional pricing may therefore be a promising intervention to reduce alcohol consumption. However, future research is needed to understand the effect of proportional pricing on alcohol consumption in a naturalistic setting.

## AUTHOR CONTRIBUTIONS

**Inge Kersbergen:** Conceptualization; methodology; formal analysis; writing—original draft; writing—review and editing; funding acquisition. **Amber Copeland:** Methodology; writing—review and editing. **Rob Pryce:** Methodology; writing—review and editing. **Petra Meier:** Supervision; writing—review and editing. **Matt Field:** Supervision; writing—review and editing.

## DECLARATION OF INTERESTS

We have no known conflicts of interest to disclose.

## Supporting information


**Table S1.** Mixed linear regression models showing the effect of proportional pricing, trial type (study 2), AUDIT scores and their interaction on alcohol units purchased. Trials were nested within individuals. Sensitivity analysis including trials in which participants purchased no alcohol.
**Table S2.** Mixed linear regression models showing the effect of trial presentation order on alcohol units purchased. Trials were nested within individuals.
**Table S3.** Mixed linear regression models showing the effect of presentation order, proportional pricing, trial type (study 2), AUDIT scores and their interaction on alcohol units purchased. Trials were nested within individuals.
**Table S4.** Mixed linear regression models showing the association between the price increase under proportional pricing (compared to standard pricing) and the increase in alcohol purchasing under proportional pricing (compared to standard pricing). Brands within trials were nested within individuals.

## Data Availability

All anonymized data are available to researchers upon request at 10.15131/shef.data.22502977. The analysis code is openly available at 10.15131/shef.data.23295179. The materials are available to researchers upon request at 10.15131/shef.data.22498969.

## Footnotes

* For products in the size and quantity difference trial type, this refers to the product that had the highest price per litre in the standard pricing condition.
