# Supplementary material for: The effect of proportional pricing on alcohol purchasing in two online experiments
Source: Addiction. 2024 Dec 3;120(5):860–70. doi: 10.1111/add.16723 (PMC11986287; doi:10.1111/add.16723)
Supplement: Supplementary file 1 — Table S1. Mixed linear regression models showing the effect of proportional pricing, trial type (study 2), AUDIT scores and their interaction on alcohol units purchased. Trials were nested within individuals. Sensitivity analysis including trials in which participants purchased no alcohol. Table S2. Mixed linear regression models showing the effect of trial presentation order on alcohol units purchased. Trials were nested within individuals. Table S3. Mixed linear regression models showing the effect of presentation order, proportional pricing, trial type (study 2), AUDIT scores and their interaction on alcohol units purchased. Trials were nested within individuals. Table S4. Mixed linear regression models showing the association between the price increase under proportional pricing (compared to standard pricing) and the increase in alcohol purchasing under proportional pricing (compared to standard pricing). Brands within trials were nested within individuals. [file ADD-120-860-s001.docx]

**Quota sampling**

| Study | Drink type | Weekly alcohol consumption | Recruited n |
| --- | --- | --- | --- |
| Study 1 | Lager | 1-13 UK units per week | 35 |
|  |  | 14+ units per week | 35 |
|  | Wine | 1-13 UK units per week | 35 |
|  |  | 14+ units per week | 35 |
|  | Vodka | 1-13 UK units per week | 35 |
|  |  | 14+ units per week | 35 |
| Study 2 | Lager | 1-13 UK units per week | 45 |
|  |  | 14+ units per week | 45 |

**Sensitivity analyses**

| **Table S1.** Mixed linear regression models showing the effect of proportional pricing, trial type (study 2), AUDIT scores and their interaction on alcohol units purchased. Trials were nested within individuals. Sensitivity analysis including trials in which participants purchased no alcohol. | | | | | | | | |
| --- | --- | --- | --- | --- | --- | --- | --- | --- |
|  | Study 1 | | | | Study 2 | | | |
|  | Overall | Lager | Wine | Vodka | All trials | Size difference only | Quantity difference only | Size and quantity difference |
| *Variables* | *B (95% CI)* | *B (95% CI)* | *B (95% CI)* | *B (95% CI)* | *B (95% CI)* | *B (95% CI)* | *B (95% CI)* | *B (95% CI)* |
| Intercept | 7.89 ^***^ (4.36, 11.42) | 3.04  (-0.65, 6.72) | 6.35 ^***^ (3.14, 9.56) | 20.66 ^***^ (14.06, 27.26) | 10.21 ^**^ (3.88, 16.55) | 7.68 ^*^ (0.92, 14.44) | 14.87 ^***^ (8.42, 21.32) | 10.21 ^**^ (3.99, 16.42) |
| Proportional pricing (Reference: Standard pricing) | -1.39 ^***^ (-2.11, -0.67) | 0.02  (-0.65, 0.68) | -0.41  (-1.17, 0.35) | -4.52 ^***^ (-6.45, -2.59) | 1.44  (-0.78, 3.66) | 0.81  (-0.56, 2.19) | -2.65 ^*^ (-5.21, -0.10) | 1.45  (-0.86, 3.75) |
| AUDIT | 0.86 ^***^ (0.58, 1.14) | 0.71 ^***^ (0.39, 1.03) | 0.43 ^**^ (0.17, 0.70) | 0.83 ^***^ (0.36, 1.30) | 0.71 ^*^ (0.16, 1.25) | 0.83 ^**^ (0.25, 1.41) | 0.50  (-0.05, 1.06) | 0.71 ^**^ (0.18, 1.24) |
| AUDIT x Proportional pricing (Reference: Standard pricing) | -0.03  (-0.09, 0.03) | -0.01  (-0.07, 0.05) | -0.14 ^***^ (-0.20, -0.08) | 0.11  (-0.03, 0.25) | -0.12  (-0.31, 0.07) | -0.08  (-0.20, 0.04) | 0.17  (-0.05, 0.39) | -0.12  (-0.32, 0.08) |
| Size difference only |  |  |  |  | -2.53 ^*^ (-4.75, -0.31) |  |  |  |
| Quantity difference only |  |  |  |  | 4.75 ^***^ (2.52, 6.98) |  |  |  |
| Size difference only x Proportional pricing |  |  |  |  | -0.62  (-3.76, 2.51) |  |  |  |
| Quantity difference only x Proportional pricing |  |  |  |  | -4.15 ^**^ (-7.31, -1.00) |  |  |  |
| Size difference only x AUDIT |  |  |  |  | 0.12  (-0.07, 0.31) |  |  |  |
| Quantity difference only x AUDIT |  |  |  |  | -0.21 ^*^ (-0.40, -0.02) |  |  |  |
| Size difference only x Proportional pricing x AUDIT |  |  |  |  | 0.04  (-0.23, 0.31) |  |  |  |
| Quantity difference only x Proportional pricing x AUDIT |  |  |  |  | 0.29 ^*^ (0.02, 0.56) |  |  |  |
| **Random Effects** | | | | | | | | |
| Within-cluster variance (σ^2­^) | 106.79 | 30.28 | 38.64 | 250.04 | 133.40 | 51.43 | 173.38 | 143.79 |
| Intercept variance (τ_00_) | 169.27 | 61.15 | 45.20 | 187.22 | 204.72 | 243.38 | 208.55 | 195.32 |
| Intraclass Correlation Coefficient | 0.61 | 0.67 | 0.54 | 0.43 | 0.61 | 0.83 | 0.55 | 0.58 |
| N participants | 207 | 70 | 68 | 69 | 90 | 90 | 90 | 90 |
| N observations | 12358 | 4183 | 4056 | 4119 | 5354 | 1798 | 1768 | 1788 |
| Marginal R^2^ / Conditional R^2^ | 0.098 / 0.651 | 0.154 / 0.720 | 0.069 / 0.571 | 0.086 / 0.477 | 0.047 / 0.624 | 0.063 / 0.836 | 0.029 / 0.559 | 0.038 / 0.592 |
| ** p<0.05   ** p<0.01   *** p<0.001* | | | | | | | | |

| **Table S2.** Mixed linear regression models showing the effect of trial presentation order on alcohol units purchased. Trials were nested within individuals. | | | | | | | | |
| --- | --- | --- | --- | --- | --- | --- | --- | --- |
|  | Study 1 | | | | Study 2 | | | |
|  | Overall | Lager | Wine | Vodka | All trials | Size difference only | Quantity difference only | Size and quantity difference |
| Variables | B (95% CI) | B (95% CI) | B (95% CI) | B (95% CI) | B (95% CI) | B (95% CI) | B (95% CI) | B (95% CI) |
| Intercept | 19.00 ^***^ (17.04, 20.96) | 11.30 ^***^ (9.25, 13.35) | 12.99 ^***^ (11.42, 14.55) | 32.65 ^***^ (29.24, 36.07) | 21.14 ^***^ (17.81, 24.46) | 19.25 ^***^ (15.75, 22.74) | 22.88 ^***^ (19.24, 26.52) | 21.48 ^***^ (17.98, 24.98) |
| Order | 0.00  (-0.01, 0.01) | -0.00  (-0.01, 0.01) | 0.00  (-0.01, 0.01) | 0.01  (-0.02, 0.03) | -0.04 ^***^ (-0.05, -0.02) | -0.03 ^***^ (-0.05, -0.01) | -0.03  (-0.06, 0.00) | -0.05 ^**^ (-0.07, -0.02) |
| **Random Effects** | | | | | | | | |
| Within-cluster variance (σ^2­^) | 86.09 | 25.01 | 25.85 | 198.03 | 105.03 | 32.42 | 120.80 | 108.55 |
| Intercept variance (τ_00_) | 199.76 | 74.45 | 40.51 | 193.87 | 250.74 | 268.64 | 282.69 | 260.45 |
| Intraclass Correlation Coefficient | 0.70 | 0.75 | 0.61 | 0.49 | 0.70 | 0.89 | 0.70 | 0.71 |
| N participants | 206 | 70 | 67 | 69 | 90 | 87 | 90 | 90 |
| N observations | 10191 | 3592 | 3020 | 3579 | 4765 | 1574 | 1589 | 1602 |
| Marginal R^2^ / Conditional R^2^ | 0.000 / 0.699 | 0.000 / 0.749 | 0.000 / 0.610 | 0.000 / 0.495 | 0.001 / 0.705 | 0.001 / 0.892 | 0.001 / 0.701 | 0.002 / 0.706 |
| ** p<0.05   ** p<0.01   *** p<0.001* | | | | | | | | |

| **Table S3.** Mixed linear regression models showing the effect of presentation order, proportional pricing, trial type (study 2), AUDIT scores and their interaction on alcohol units purchased. Trials were nested within individuals. | | | | | | | | |
| --- | --- | --- | --- | --- | --- | --- | --- | --- |
|  | Study 1 | | | | Study 2 | | | |
|  | Overall | Lager | Wine | Vodka | All trials | Size difference only | Quantity difference only | Size and quantity difference |
| Variables | B (95% CI) | B (95% CI) | B (95% CI) | B (95% CI) | B (95% CI) | B (95% CI) | B (95% CI) | B (95% CI) |
| Intercept | 11.89 ^***^ (8.16, 15.62) | 5.85 ^**^ (2.12, 9.58) | 9.19 ^***^ (6.15, 12.22) | 27.43 ^***^ (20.67, 34.19) | 17.85 ^***^ (10.49, 25.21) | 13.61 ^***^ (6.36, 20.86) | 20.93 ^***^ (13.12, 28.75) | 18.08 ^***^ (10.52, 25.65) |
| Proportional pricing (Reference: Standard pricing) | -1.92 ^**^ (-3.36, -0.49) | -0.45  (-1.77, 0.86) | -1.18  (-2.60, 0.24) | -5.09 ^**^ (-8.87, -1.31) | 0.38  (-3.88, 4.65) | -0.08  (-2.46, 2.30) | -1.04  (-5.55, 3.47) | 0.67  (-3.78, 5.12) |
| AUDIT | 0.71 ^***^ (0.42, 1.00) | 0.54 ^**^ (0.21, 0.86) | 0.40 ^**^ (0.16, 0.65) | 0.56 ^*^ (0.07, 1.04) | 0.39  (-0.24, 1.02) | 0.58  (-0.04, 1.19) | 0.17  (-0.50, 0.84) | 0.40  (-0.25, 1.04) |
| Order | -0.02  (-0.05, 0.01) | -0.04 ^**^ (-0.07, -0.01) | -0.00  (-0.03, 0.02) | -0.02  (-0.09, 0.05) | -0.10 ^*^ (-0.18, -0.02) | -0.07 ^**^ (-0.12, -0.02) | -0.07  (-0.16, 0.01) | -0.10 ^*^ (-0.18, -0.01) |
| AUDIT x Proportional pricing (Reference: Standard pricing) | 0.06  (-0.06, 0.17) | 0.08  (-0.03, 0.19) | -0.00  (-0.12, 0.11) | 0.17  (-0.10, 0.43) | -0.15  (-0.51, 0.21) | -0.06  (-0.26, 0.14) | 0.15  (-0.23, 0.53) | -0.19  (-0.57, 0.18) |
| **Order x Proportional pricing (Reference: Standard pricing)** | **0.03  (-0.01, 0.07)** | **0.02  (-0.02, 0.05)** | **0.03  (-0.01, 0.07)** | **0.06  (-0.05, 0.16)** | **0.02  (-0.10, 0.13)** | **0.01  (-0.06, 0.08)** | **-0.05  (-0.18, 0.07)** | **0.01  (-0.11, 0.13)** |
| AUDIT x Order | 0.00  (-0.00, 0.00) | 0.00 ^***^ (0.00, 0.01) | 0.00  (-0.00, 0.00) | 0.00  (-0.00, 0.01) | 0.00  (-0.00, 0.01) | 0.00  (-0.00, 0.01) | 0.01  (-0.00, 0.01) | 0.00  (-0.00, 0.01) |
| AUDIT x Order x Proportional pricing (Reference: Standard pricing) | -0.00  (-0.01, 0.00) | -0.00  (-0.01, 0.00) | -0.00 ^*^ (-0.01, -0.00) | -0.00  (-0.01, 0.00) | 0.00  (-0.01, 0.01) | 0.00  (-0.01, 0.01) | 0.00  (-0.01, 0.01) | 0.00  (-0.01, 0.01) |
| Size difference only |  |  |  |  | -3.79  (-8.03, 0.45) |  |  |  |
| Quantity difference only |  |  |  |  | 2.99  (-1.30, 7.28) |  |  |  |
| Size difference only x Proportional pricing |  |  |  |  | -1.88  (-7.85, 4.09) |  |  |  |
| Quantity difference only x Proportional pricing |  |  |  |  | -1.79  (-7.73, 4.14) |  |  |  |
| Size difference only x AUDIT |  |  |  |  | 0.15  (-0.20, 0.50) |  |  |  |
| Quantity difference only x AUDIT |  |  |  |  | -0.17  (-0.53, 0.19) |  |  |  |
| Size difference only x Order |  |  |  |  | 0.01  (-0.10, 0.13) |  |  |  |
| Quantity difference only x Order |  |  |  |  | 0.03  (-0.09, 0.14) |  |  |  |
| Size difference only x Proportional pricing x AUDIT |  |  |  |  | 0.20  (-0.30, 0.71) |  |  |  |
| Quantity difference only x Proportional pricing x AUDIT |  |  |  |  | 0.30  (-0.20, 0.80) |  |  |  |
| Size difference only x Proportional pricing x Order |  |  |  |  | 0.04  (-0.13, 0.20) |  |  |  |
| Quantity difference only x Proportional pricing x Order |  |  |  |  | -0.06  (-0.22, 0.10) |  |  |  |
| Size difference only x AUDIT x Order |  |  |  |  | 0.00  (-0.01, 0.01) |  |  |  |
| Quantity difference only x AUDIT x Order |  |  |  |  | 0.00  (-0.01, 0.01) |  |  |  |
| Size difference only x Proportional pricing x AUDIT x Order |  |  |  |  | -0.01  (-0.02, 0.01) |  |  |  |
| Quantity difference only x Proportional pricing x AUDIT x Order |  |  |  |  | -0.00  (-0.01, 0.01) |  |  |  |
| **Random Effects** | | | | | | | | |
| Within-cluster variance (σ^2­^) | 85.62 | 24.87 | 25.20 | 196.32 | 101.91 | 32.24 | 119.57 | 108.12 |
| Intercept variance (τ_00_) | 176.35 | 59.73 | 35.77 | 173.63 | 242.22 | 255.59 | 274.50 | 253.50 |
| Intraclass Correlation Coefficient | 0.67 | 0.71 | 0.59 | 0.47 | 0.70 | 0.89 | 0.70 | 0.70 |
| N participants | 206 | 70 | 67 | 69 | 90 | 87 | 90 | 90 |
| N observations | 10191 | 3592 | 3020 | 3579 | 4765 | 1574 | 1589 | 1602 |
| Marginal R^2^ / Conditional R^2^ | 0.086 / 0.701 | 0.155 / 0.752 | 0.091 / 0.624 | 0.056 / 0.499 | 0.036 / 0.714 | 0.046 / 0.893 | 0.021 / 0.703 | 0.021 / 0.707 |
| ** p<0.05   ** p<0.01   *** p<0.001* | | | | | | | | |

**Exploratory analyses**

| **Table S4.** Mixed linear regression models showing the association between the price increase under proportional pricing (compared to standard pricing) and the increase in alcohol purchasing under proportional pricing (compared to standard pricing). Brands within trials were nested within individuals. | | | | | | | | |
| --- | --- | --- | --- | --- | --- | --- | --- | --- |
|  | Study 1 | | | | Study 2 | | | |
|  | Overall | Lager | Wine | Vodka | All trials | Size difference only | Quantity difference only | Size and quantity difference |
| Variables | B (95% CI) | B (95% CI) | B (95% CI) | B (95% CI) | B (95% CI) | B (95% CI) | B (95% CI) | B (95% CI) |
| Intercept | -0.27  (-0.56, 0.02) | 0.19 ^*^ (0.02, 0.35) | 0.73 ^*^ (0.13, 1.33) | -0.44  (-1.23, 0.36) | 0.77 ^***^ (0.38, 1.17) | 1.31 ^***^ (0.61, 2.01) | 3.87 ^***^ (1.96, 5.78) | 1.29 ^***^ (0.62, 1.97) |
| Price increase under proportional pricing | -0.34 ^***^ (-0.41, -0.28) | -0.95 ^***^ (-1.44, -0.46) | -1.04 ^***^ (-1.28, -0.79) | -0.31 ^***^ (-0.41, -0.20) | -0.74 ^***^ (-0.98, -0.51) | -2.71 ^***^ (-3.89, -1.52) | -1.70 ^***^ (-2.37, -1.02) | -1.75 ^***^ (-2.52, -0.99) |
| Random effects | | | | | | | | |
| Within-cluster variance (σ^2­^) | 88.98 | 10.74 | 32.77 | 214.23 | 89.50 | 68.71 | 102.72 | 91.78 |
| Intercept variance (τ_00_) | 1.87 | 0.07 | 1.62 | 3.86 | 0.00 | 0.00 | 3.26 | 0.00 |
| Intraclass Correlation Coefficient | 0.02 | 0.01 | 0.05 | 0.02 |  |  | 0.03 |  |
| N participants | 205 | 69 | 67 | 69 | 89 | 86 | 89 | 89 |
| N observations | 10580 | 3578 | 3299 | 3703 | 4795 | 1584 | 1598 | 1613 |
| Marginal R^2^ / Conditional R^2^ | 0.013 / 0.033 | 0.004 / 0.010 | 0.020 / 0.066 | 0.009 / 0.026 | 0.008 / NA | 0.013 / NA | 0.015 / 0.045 | 0.012 / NA |
| ** p<0.05   ** p<0.01   *** p<0.001* | | | | | | | | |
